# Vestibular physiology and function in zebrafish

**DOI:** 10.3389/fcell.2023.1172933

**Published:** 2023-04-18

**Authors:** Selina Baeza-Loya, David W. Raible

**Affiliations:** Virginia Merrill Bloedel Hearing Research Center, Department of Otolaryngology-HNS and Biological Structure, University of Washington, Seattle, WA, United States

**Keywords:** zebrafish, vestibular, inner ear, statoacoustic ganglion, vestibular reflex

## Abstract

The vestibular system of the inner ear provides information about head motion and spatial orientation relative to gravity to ensure gaze stability, balance, and postural control. Zebrafish, like humans, have five sensory patches per ear that serve as peripheral vestibular organs, with the addition of the lagena and macula neglecta. The zebrafish inner ear can be easily studied due to its accessible location, the transparent tissue of larval fish, and the early development of vestibular behaviors. Thus, zebrafish are an excellent model for studying the development, physiology, and function of the vestibular system. Recent work has made great strides to elucidate vestibular neural circuitry in fish, tracing sensory transmission from receptors in the periphery to central computational circuits driving vestibular reflexes. Here we highlight recent work that illuminates the functional organization of vestibular sensory epithelia, innervating first-order afferent neurons, and second-order neuronal targets in the hindbrain. Using a combination of genetic, anatomical, electrophysiological, and optical techniques, these studies have probed the roles of vestibular sensory signals in fish gaze, postural, and swimming behaviors. We discuss remaining questions in vestibular development and organization that are tractable in the zebrafish model.

## Introduction

The peripheral vestibular organs sense head acceleration through space and tilt with respect to gravity. Signals carrying information about linear and rotational head motions are integrated into postural, autonomic, and ocular reflexes necessary for maintaining posture, orientation, and a stable visual world. The need to detect motion through space, the features of the inner ear structures that sense motion, and fundamental vestibular neural circuitry that encodes sensory information is conserved across species, from fish to birds to mice to humans. Vestibular dysfunction is common in humans, increases with age, and is a major contributor to falls and resulting morbidity (Agrawal et al., 2009).

Comprehensive dissection of vestibular neural circuitry is difficult due to many different challenges. The inner ear tissues are delicate, complex in their organization, and difficult to access within the confines of the temporal bone in many mature animals. Hair cells, the sensory receptors that convert motion into neural signals, are susceptible to injury and do not regenerate in mammals. Vestibular sensory inputs have high dimensionality; organisms move in three dimensions in linear space in addition to undergoing angular accelerations. These complex inputs are integrated through a multitude of sensory, motor, and cognitive computations, the dissection of which becomes difficult in more physiologically complex animal models (i.e., increased number of limbs, upright postural reflexes, etc.). As such, vestibular physiology and circuits in the periphery, central nuclei, and forebrain integration, as well as contributions to cognitive functions such as memory and spatial awareness, are poorly understood and understudied.

Zebrafish, as a model organism, have unique features that circumvent these challenges. First, zebrafish are transparent as larvae and develop quickly. Zebrafish inner ears develop soon after fertilization; hair cells form at 24 h post fertilization (hpf) and the vestibular and auditory systems are functional at 5 days post fertilization (dpf) (Hussain et al., 2022). This quick developmental timeline allows for direct anatomical, electrophysiological, and optical research into the ear (Bagnall and Schoppik, 2018; Hussain et al., 2022). Second, zebrafish are genetically accessible, which allows for the generation of mutants that label or perturb specific structures in the ear, vestibular nerve, or nuclei that can result in observable vestibular phenotypes. Relatedly, and third, zebrafish have a simple anatomy where the development of swimming and natural balancing behaviors can be observed. Thus, as technologies and tools have progressed, research in vestibular circuitry in zebrafish has advanced significantly in recent years. In this review, we will describe the anatomy and physiology of the zebrafish inner ear and highlight recent published work that illuminates functional vestibular circuits at the level of first- and second-order vestibular neurons.

### Inner ear morphology in zebrafish

**FIGURE 1:**
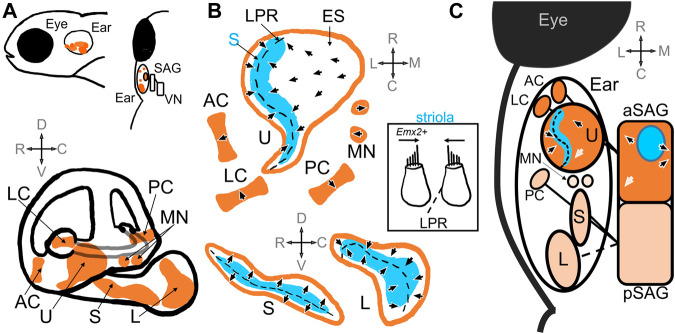
Schematic of the zebrafish inner ear. **(A)** Top panel includes side and top-down views that demonstrate relative location of the zebrafish inner ear posterior to the eye. The ear is innervated by the statoacoustic ganglion (SAG), which synapses on vestibular nuclei (VN) in the hindbrain. The bottom panel shows the relative positioning of the utricle (U), saccule (S) and lagena (L) otolith maculae, and the anterior (AC), posterior (PC), and lateral (LC) cristae, as well as the macula neglecta (MN) within the inner ear (Platt, 1993; Schoppik et al., 2017). **(B)** Diagrams of the sensory epithelia show the position of the striola (S, blue) and extrastriola (ES) zones in the utricle, saccule, and lagena. Top, end organs in the horizontal plane; Bottom end organs in the saggital plane. Arrow heads indicate the general polarity of the hair cells in the epithelia, which change direction along the line of polarity reversal (LPR, dashed black line) in the otolith maculae (Platt, 1993; Liu et al., 2022; Shi et al., 2023). There is no LPR in the cristae or macula neglecta. Inset: A patch of tissue taken from the striola in the utricle along the LPR that includes hair cells that would have opposite polarities (Platt, 1993). Hair cells in the lateral utricle express emx2, which triggers changes in polarities during hair cell development (Jiang et al., 2017). **(C)** A schematic showing a top-down view of the inner ear and SAG. The utricle, anterior and lateral cristae are innervated by the anterior compartment of the SAG (aSAG): the striolar zone (blue) of the utricle is innervated by SAG neurons whose cell bodies reside in the interior of the aSAG (blue circle) (Liu et al., 2022). The topography of the polarities of the hair cells in the utricle is preserved in the SAG (arrows): e.g., caudally located SAG neurons have caudal directional tuning informed by hair cells with caudal polarities (white arrows) (Tanimoto et al., 2022). The saccule, posterior cristae, and possibly the lagena are innervated by the posterior compartment of the SAG (pSAG); the organization of the pSAG is unknown. It is unknown which compartment innervates the macula neglecta.

The zebrafish inner ear contains vestibular end organs that also serve in hearing (Figure 1A). In teleost fishes, as in other vertebrates, the vestibular organs are divided into a gravity receptor system and an angular acceleration receptor system. The gravity receptor system detects linear acceleration, and consists of utricular, saccular, and lagenar sensory epithelia (also known as maculae) (Platt, 1993; Moorman, 2001) (Figure 1A). In teleost fishes, each macula has a corresponding otolith, a calcium carbonate stone that deflects mechanosensitive hair cells in response to movement from inertial forces, and are thus called the otolith organs (Figure 1B). The angular acceleration system consists of three orthogonal semicircular ducts, each with an ampulla containing a sensory epithelium (known as a crista) where hair cells are stimulated via endolymph flow (Platt, 1993; Moorman, 2001) (Figure 1B). Hair cells in the end organs are innervated by first-order vestibular afferent neurons, known as statoacoustic ganglion (SAG) neurons. These bipolar neurons make up the VIII cranial nerve and carry information to various nuclei in the rhombomeres of the hindbrain (Andermann et al., 2002; Whitfield et al., 2002) (Figure 1A). Thus, the main components of the zebrafish vestibular inner ear are comparable to other model animal systems (Platt and Popper, 1981; Moorman, 2001; Bever and Fekete, 2002; Ladich and Schulz-Mirbach, 2016; Jiang et al., 2017).

The sensory epithelia and afferent neurons of the zebrafish inner ear arise from the otic placode, an ectodermal thickening, which is converted into the mature organs (Torres and Giráldez, 1998; Alsina and Whitfield, 2017). Evidence indicates asymmetrical and asynchronous development of the otolith organs, suggesting independent regulation. The utricle and the saccule are the first sensory epithelia to develop, along with neuroblasts that form the neurons of the SAG (Haddon and Lewis, 1996). The utricle begins to develop hair cells at 27 hpf while hair cells in the saccule develop as late as 34 hpf. One possible cause is the asynchronous regulation of two atonal homologs, *atoh1a* and *atoh1b*, that encode for conserved bHLH transcription factors necessary for hair cell development (Millimaki et al., 2007; Sapède and Pujades, 2010). There is evidence indicating that the development of the saccule may also be influenced by Hedgehog (Hh) signaling upstream of *atoh1b* (Hammond et al., 2003; Sapède and Pujades, 2010), a signaling factor known to regulate cochlear development in mammals (Riccomagno et al., 2002). For an in-depth review on the development of the zebrafish inner ear, see Abbas and Whitfield, 2010.

The development of the zebrafish inner ear is well documented and follows a stereotyped timeline (Haddon and Lewis, 1996; Bang et al., 2001; Bever and Fekete, 2002; Higgs et al., 2003). While the utricle and saccule sensory maculae and associated otoliths are established during the second day of development, sensory epithelia of the cristae begin formation at 3 dpf and the lagena and associated otolith by 15–17 dpf. An additional sensory patch, the macula neglecta, is apparent by 17 dpf at a location between the utricle and saccule. Like the cristae, the macula neglecta has no associated otolith. Structural aspects of the inner ear also continue to mature over the larval and juvenile stages. The dorsal or upper part of the ear (pars superior), consisting of semicircular canals and utricle, is formed first, with the basic configuration apparent by 5 dpf. The ventral or lower part of the ear (pars inferior) develops later, with the saccular pouch beginning to protrude at 5 dpf and laginar pouch at 17 dpf. By the end of the juvenile stage, at 30 dpf, the ear has reached its adult configuration. However, the ear will continue to grow five times its size during adulthood, with a ten-fold increase in hair cell number in each sensory patch (Bang et al., 2001). How functional integrity is maintained during growth is currently unknown.

The semicircular canals are major vestibular sensors in adult animals (Riley and Moorman, 2000; Ladich and Schulz-Mirbach, 2016; Bagnall and Schoppik, 2018). The cristae of the semicircular canals are largely conserved across fishes, making them the most strongly conserved sensory epithelia (Ladich and Schulz-Mirbach, 2016). During head rotations, a gelatinous structure within the ampulla known as the cupula is deformed by endolymph flow during motion, deflecting hair cells in the cristae. However, at larval stages (5–7 dpf), semicircular canals are too small to sense motion (Beck et al., 2004; Lambert et al., 2008). Semicircular canals may not be functional until past 30 dpf when the canals are sufficiently large enough for inertial forces to induce endolymph flow (Bever and Fekete, 2002; Beck et al., 2004). The delay in semicircular development implicates the utricle as the main vestibular organ in larval zebrafish, especially during crucial periods for behavioral development (Riley and Moorman, 2000; Bagnall and Schoppik, 2018).

The utricle shows minimal variation in shape and orientation across fish and other vertebrates (Platt and Popper, 1981; Ladich and Schulz-Mirbach, 2016). In addition to being the first epithelium to develop, it is also vital for larval survival (Riley and Moorman, 2000). Bilateral utricle deficient mutants will die by 16 dpf; preservation of the utricle on one side significantly improves survival rates while bilateral saccular deficient mutants do not experience decreased survival (Riley and Moorman, 2000). In larval and adult zebrafish, the utricle is essential for detecting gravity. When a utricle is disrupted via genetic manipulation or physical ablation, fish fail to detect gravity, leading to a loss of gaze compensation and altered balance (Riley and Moorman, 2000; Kwak et al., 2006; Mo et al., 2010). Mutants swam in circles, roll, or spiral, and could not balance upright. However, there is evidence to suggest that vestibular circuits remain plastic after early development: mutant zebrafish lacking a functional *otegelin* allele (*otog* ^−/−^ mutants) have delayed utricular otolith development until around 14 dpf, well after initial development of vestibular circuits, but still learn to balance and swim normally relative to gravity (Bagnall and McLean, 2014; Roberts et al., 2017). Given the function of the utricle as the main vestibular organ in larval and adult zebrafish, much research on vestibular function and development has focused on this organ (ex., Favre-Bulle et al., 2017; Liu et al., 2020; Jia and Bagnall., 2022; Liu et al., 2022; Tanimoto et al., 2022).

Although all three maculae are involved in sound detection (Yao et al., 2016; Privat et al., 2019; Favre-Bulle et al., 2020), the saccule has been implicated as the main hearing organ in zebrafish (Schuck and Smith, 2009; Breitzler et al., 2020). At larval stages, the saccule responds primarily to auditory stimuli, possibly due to the large size of its otolith (Inoue et al., 2013; Yao et al., 2016; Lara et al., 2022). Studies done in the goldfish indicate that the saccule has tonotopic organization, where caudal to rostral regions are sensitive to low to high frequencies, respectively (Smith et al., 2011). Given the similarities between goldfish and zebrafish otolith organs (Platt, 1993), an analogous organization is possible in zebrafish. Some studies have shown that noise overexposure of specific tones results in hair cell damage at defined regions of zebrafish saccule, consistent with tonotopic organization (Schuck and Smith, 2009; Breitzler et al., 2020; Han et al., 2022). Many fishes are also thought to use the lagena as part of their auditory system (Ladich and Schulz-Mirbach, 2016), although zebrafish lagenar hair cells do not begin to develop until 15 dpf, well after hearing onset (Bever and Fekete, 2002). In sharks the macula neglecta is thought to respond to sound vibration (Fay et al., 1974), but has not been implicated in teleost fishes. The relative contributions of each zebrafish end organ to both vestibular and auditory senses remain unresolved.

### Vestibular hair cells

Hair cells are the mechanoreceptors that capture sound in the cochlea, head movements in the vestibular organs, and water movements in the fish lateral line. These stimuli are detected through deflection of an apical hair bundle. The hair bundle is a brush of actin-based stereocilia aligned in rows of graded heights, with tallest rows adjacent to a primary microtubule-based cilium known as the kinocilium (Figure 1B insert). This asymmetric morphology enables hair cells to respond to mechanical stimuli in a directional manner. When the bundle is deflected in the direction of the kinocilium, extracellular links between rows of stereocilia open mechanosensitive channels at stereocilia tips. Both proper morphology and orientation of hair bundles within sensory organs are critical for organ function. For in-depth reviews of zebrafish hair cells and associated methods, in both inner ear and lateral line, see Abbas and Whitfield, 2010, Nicolson, 2017, Pickett and Raible, 2019 and Hussain et al., 2022.

The orientation of the hair bundles dictates the direction selectivity, or polarity, of the hair cell (Figure 1B). Within the maculae, hair cells near each other tend to have similar direction selectivity relative to those further away (Liu et al., 2022). The general orientation of hair bundles changes across the line of polarity reversal (LPR), where adjacent hair cells have opposite polarity (Figure 1B). When one side of the macula is deflected in its preferred direction, those hair cells are depolarized while hair cells on the opposite side of the LPR are hyperpolarized. In the utricle the LPR lies toward the lateral edge, while in the saccule and lagena it is located medially (Platt, 1993) (Figure 1B). In the utricle and the lagena the direction of hair cell sensitivity faces the LPR, while in the saccule the orientation face away (Figure 1B). Therefore, the lateral edge of the utricle has the same polarity as the medial saccule. In the cristae and macula neglecta, the hair cells have uniform directional orientation and therefore no LPR, but the epithelia are oriented in different directions (Figure 1B). Opposite directional selectivity allows the sensory epithelia to detect stimuli in different directions.

Polarity reversal in hair cells is crucial for the detection of motion in opposite directions within a macula. Differences in polarity has been shown to depend on the expression of the conserved homeobox transcription factor Emx2 in hair cells (Figure 1B inset). Emx2 is found in a subset of hair cells at the lateral edge of the utricle and the medial side of the saccule and is necessary for the differences in polarity in the hair cells in these regions (Jiang et al., 2017; Kindt et al., 2021). Jiang et al., 2017 showed that mutation of zebrafish *emx2* resulted in absence of polarity reversal in utricle, saccule, and lateral line. *Emx2* mutant fish had utricles with no LPR and all hair cells oriented in one direction, which was also observed in mice and chicks. Ectopic expression of *emx2* in all hair cells under a cell-specific promoter also resulted in loss of the LPR but exhibited polarities in the opposite direction compared to loss-of-function mutants. Hair cells subsequently only responded to stimulation in one direction. These mutant larval fish also displayed abnormal swimming behaviors before dying. Thus, differences in polarity reversal are also necessary for normal swimming behaviors and survival. Elegant follow-up studies subsequently demonstrated that the polarization of Emx2 also polarized distribution of a G-protein-coupled receptor, GPR156-Gαi, which triggered reversal in hair cell orientation in zebrafish lateral line and in mouse vestibular and auditory organs (Kindt et al., 2021). Thus, molecular mechanisms that trigger polarity reversal are evolutionarily conserved and necessary for normal vestibular function.

In addition to their directional selectivity, hair cells can also be characterized as either striolar or extrastriolar based on their spatial location within the maculae (Figure 1B). The striolar and extrastriolar regions are anatomically defined landmarks that coincide with differences in hair cell morphology and afferent innervation. In mice, monkeys, and frogs, striolar hair cells are often Type I gourd-shaped hair cells that are innervated by cup-like calyx afferent endings. Extrastriolar hair cells are often Type II hair cells, which are cylindrical in shape and have bouton afferents, although some Type I cells are also found in the periphery (Fernandez and Goldberg, 1976; Baird and Lewis, 1986; Songer and Eatock, 2013). Fish do not have morphologically distinct Type I hair cells as seen in amniotes, although enlarged quasi-calyx afferent endings has been observed in goldfish cristae (Lanford and Popper, 1996). Thus, zebrafish inner ear afferents are often described as Type II-like in their morphology (Liu et al., 2022). However as discussed below, zebrafish hair cells share no clear molecular similarity with either mammalian Type I or Type II hair cells. Additional morphological, genetic, and functional studies are needed to define distinct populations of hair cells in zebrafish maculae.

In zebrafish, striolar hair cells can be characterized by the relative length of the kinocilium to the tallest stereocilia (Platt, 1993). In the adult zebrafish utricle, striolar hair cells have kinocilia and stereocilia lengths approximately 5 μm whereas extrastriolar hair cells have longer kinocilia (6–8 μm) and shorter stereocilia (2-3 μm) (Platt, 1993; Liu et al., 2022). Utricular striolar hair cells straddle the LPR while extrastriolar hair cells populate the rest of the maculae (Liu et al., 2022) (Figure 1B). In rodents, utricular hair cells develop in a spatiotemporal sequence, radiating from the striola out to the periphery (Burns et al., 2012; Jiang et al., 2017; Warchol et al., 2019). Liu et al., 2022 demonstrated that in the zebrafish, too, centrally located striolar hair cells were the most mature, as shown by longer kinocilium, greater number of synaptic ribbons, and myelinated afferents, while peripherally located extrastriolar hair cells were the most immature. The morphological and developmental characteristics of hair cells in the otolith maculae in zebrafish therefore depend on spatial location in the epithelia.

Hair cells in the adult and juvenile inner ear display diverse biophysical properties that contribute to sensory transduction (Haden et al., 2013; Olt et al., 2014). When investigated via whole-cell patch clamp electrophysiology, macular hair cells from juvenile and adult zebrafish display multiple currents that changed in expression over the course of development. In the adult lagena and saccule, striolar hair cells had different biophysical profiles from those in the extrastriola: striolar hair cells were reported to have primarily multiple voltage-gated potassium currents while extrastriolar hair cells expressed calcium-activated potassium currents, hyperpolarization-activated cyclic nucleotide-gated currents, and voltage-gated calcium currents in addition to voltage-gated potassium currents (Olt et al., 2014). The saccular extrastriolar hair cell electrophysiology is purportedly similar to that of mouse Type II vestibular hair cells, but without inward sodium currents (Eatock et al., 1998). The saccular striolar hair cell profile is likened to immature mouse cochlear inner hair cells (Marcotti et al., 2003). Utricular hair cells from both zones were reported to be more homogeneous, similar to saccular extrastriolar hair cells, and like mouse Type II hair cells in their electrophysiology. The biophysical profiles of zebrafish inner ear hair cells have a developmental timeline, are zone dependent, and are comparable to mouse vestibular hair cells.

Zebrafish striolar and extrastriolar hair cells are also functionally different, preferentially responding to different sensory inputs. Using Ca^2+^ imaging, Tanimoto et al., 2022 demonstrated that striolar hair cells preferentially responded to fast dynamic vestibular input in the form of vibrations while extrastriolar hair cells preferred slow static tilts. In the mouse, striolar Type I hair cells have several biophysical advantages for capturing high frequency stimulation (Songer and Eatock, 2013): electrical adaptation of mechanoelectrical transduction, addition of low-voltage-activated potassium channels to the hair-cell membrane, and non-quantal transmission. Even without these characteristics, striolar zebrafish hair cells still preferentially respond to high frequency stimulation. However, the morphological and biophysical mechanisms that drive differences in hair cell responses are unknown. Zebrafish hair cells in the striola and extrastriola therefore also vary in their functional characteristics.

Zebrafish inner ear hair cells vary in their molecular signatures (Barta et al., 2018; Hoffman et al., 2018; Shi et al., 2023). Using single-cell RNA sequencing, Shi et al., 2023 showed that hair cells from the inner ear have distinct genetic profiles. Using unsupervised clustering, hair cells from the maculae and cristae were distinctly separated, as were striolar and extrastriolar cells. Striolar hair cells expressed *cabp2b+*, which overlaps with *pvalb9*, an orthologue of the mammalian striolar marker oncomodulin (Hoffman et al., 2018), while extrastriolar cells expressed *capb1b+* in a domain that is exclusive of *pvalb9* (Shi et al., 2023). Similarly, central and peripheral zones of cristae were molecularly distinct. Computational tools comparing transcriptional profiles across species revealed shared homology between zebrafish and mouse striolar and extrastriolar hair cells. However, zebrafish hair cells showed no obvious alignment with either mouse Type I or Type II hair cells. These comparisons were constrained by the limited data available for mouse vestibular hair cells, and more definitive analysis will be needed with additional study.

Further support for molecular distinction between hair cells comes from demonstrated differences in paralog genes encoding Tmc proteins that form the basis of the mechanotransduction channel. Several studies have demonstrated different functional requirements for zebrafish *tmc1*, *tmc2a* and *tmc2b* using zebrafish mutants alone and in combination (Chou et al., 2017; Chen et al., 2020; Smith et al., 2020; Zhu et al., 2021). Triple mutants lack both auditory responses as assessed by acoustic behavioral startle and microphonic measurements (Chen et al., 2020; Smith et al., 2020) and vestibular responses assessed by vestibular-induced eye movement (Smith et al., 2020). The function of *tmc1* is largely expendable for hearing (Chen et al., 2020; Smith et al., 2020). Mutations in both *tmc2a* and *tmc2b* eliminated microphonic responses (Chen et al., 2020; Smith et al., 2020), and eliminated behavioral responses to pure tone stimuli (Smith et al., 2020) but only attenuated acoustic startle in response to vibrational tap stimuli (Chen et al., 2020). Assessment of vestibular function via vestibular-induced eye movement (Smith et al., 2020) indicated *tmc1* and *tmc2a* were expendable, *tmc2b* mutants had somewhat reduced function, and that *tmc1/2b* and *tmc2a/2b* mutants have significantly reduced vestibular function relative to wildtypes or other mutant combinations. Mechanotransduction-dependent dye uptake in wildtype and mutant revealed that hair cells in distinct locations required different combinations of Tmc proteins (Smith et al., 2020; Zhu et al., 2021). In the cristae, two distinct populations of hair cells required either *tmc1/2b* or *tmc2a*, corresponding to an apical and basal layer of cells, respectively, with some exceptions in the lateral crista. In the saccule, dye uptake in the regions corresponding to the striola required tmc2a/2b while uptake in the region corresponding to the extrastriola required functional *tmc1*. Therefore, hair cells in the inner ear have differential expression in key genes that contribute to hair cell mechanotransduction, and ultimately vestibular function.

And finally, zebrafish hair cells regenerate. Zebrafish, like many fish species, have the ability to regrow hair cells in their inner ear following damage (Schuck and Smith, 2009; Liang et al., 2012; Jimenez et al., 2021; Jimenez et al., 2022). Mammalian species lose hair cells to age or injury and experience limited regeneration, resulting in permanent hearing loss and balance deficiencies (Warchol, 2011; Rubel et al., 2013). Fish and birds continuously regenerate hair cells throughout their lifespan (Warchol, 2011; Rubel et al., 2013). Thus, zebrafish hair cells can be used to probe pathways to promote hair cell survival and regeneration in the hopes of therapy development for hearing and balance disorders (Kindt and Sheets, 2018; Sheets et al., 2021).

### First-order vestibular neurons

Each hair cell is innervated by first-order vestibular neurons that convey motion and vibration information from the periphery to central nuclei in the hindbrain (Figure 1C). These afferent neurons derive from the anterior and posteromedial part of the otic vesicle from which neuroblasts start to delaminate at 22 hpf and then coalesce to form the SAG by 48 hpf (Haddon and Lewis, 1996; Sapède et al., 2012). Inactivation of Hh signaling has been shown to reduce the number of SAG neurons between 42 hpf and 3 dpf (Sapède and Pujades, 2010), indicating this period of development may be crucial for ganglion development. The ganglion itself is composed of two compartments, the neurons of which may preferentially innervate different sensory epithelia (Figure 1C). The anterior compartment innervates the utricle, the anterior and lateral cristae, and the posterior compartment innervates the saccule and posterior cristae (Figure 1C) (Sapède and Pujades, 2010; Taberner et al., 2017). Interruption of various early-expressing differentiation genes, such as Hh (Sapède and Pujades, 2010) and *short stature homeobox domain 2* (*shox2*) (Laureano et al., 2022), reduced and disorganized the SAG, and in the case of Hh, induced fibers from the compartments to extend to the wrong epithelium. Sapède and Pujades, 2010 traced SAG innervation in zebrafish embryos prior to when the lagena is developed, but given its locations in the pars inferior it is likely that the lagena too is innervated by the posterior SAG compartment. It is unknown which compartment innervates the macula neglecta. The ganglion thus develops soon after the sensory epithelia and has a broad structural organization.

Zebrafish are known to regenerate hair cells continuously throughout their lifespan, but do not necessarily regenerate afferent neurons. Studies have demonstrated that new neurons are developed in the SAG from 48 hpf well into late juvenile stages (28 dpf) (Schwarzer et al., 2020). However, new neurons are few in the adult, indicating that new hair cells regenerated in mature fish are likely innervated by preexisting neurons (Schwarzer et al., 2020). Synapse formation and regeneration has been studied extensively in the lateral line, where hair cells and their synapses are easily accessible on the external surface of the fish (Lush and Piotrowski, 2014; Pujol-Martí et al., 2014; Suli et al., 2016; Hardy et al., 2021; Holmgren et al., 2021). Here, functional hair cell/afferent synapses have been shown to depend on a complex interaction of calcium influx through voltage-gated channels, mitochondria, and synaptic ribbons in the pre- and postsynaptic densities (Sheets et al., 2011; Sheets et al., 2012; Wong et al., 2019). While little is known about afferent ending regeneration in the inner ear, it is possible that findings from the lateral line apply to the inner ear.

SAG afferents innervate the utricle in a spatiotemporal manner. Electron microscopy (EM) reconstruction in 5 dpf fish showed the earliest-born neurons innervate hair cells in the striola, where the most mature of the hair cells are located (Figure 1C) (Liu et al., 2022). As afferents are born, they innervate hair cells outward from the striola but only synapse on a single side of the LPR. The latest-born neurons synapse on the most immature hair cells on the peripheral extrastriola of the utricle (Liu et al., 2022).

Studies also indicate there is spatiotemporal organization within the SAG. EM analysis shows that earliest-born neurons are found on the lateral edge of the ganglion (Liu et al., 2022), closest to the ear. As neurons develop, they are added to form a “shell” around the earlier born neurons (Figure 1C). EM reconstruction of afferent projections suggests neurons close to each other in the SAG may have similar tuning. For example, neurons located in the caudal SAG are likely to synapse on hair cells with caudal head tilt direction selectivity, putatively resulting in caudal directional tuning (Figure 1C). Ca^2+^ imaging during vestibular stimulation in 5 dpf fish provides evidence to corroborate this hypothesis: neurons in the SAG are activated in a topographical manner in response to tilt stimuli (Tanimoto et al., 2022). Neurons located caudally in the SAG have caudal response vectors and rostrally located neurons have rostral response vectors (Figure 1C). Tanimoto et al., 2022 also showed that SAG neurons respond to largely utricular input in the form of static tilts or vibrations. Neurons responded to either tilts (40%), both tilts and vibration (10%), and or only vibrations (2%). Neurons on the outermost edge of the SAG “shell” tended to not respond at all (48%). Evidence from EM studies suggests these are likely the most immature neurons (Liu et al., 2022), which either may not be functional at this larval stage or may innervate other sensory epithelia. Thus, structural and functional studies have shown spatiotemporal functional organization of neurons within the SAG compartments.

Projections from the SAG to central vestibular targets are parallel but distinct. Studies of the sensory cranial nerves, including the SAG, suggest topographical organization of cranial nerve axonal projections (Zecca et al., 2015; Taberner et al., 2017). SAG neurons develop after trigeminal ganglion neurons but before lateral line ganglion neurons. Axonal projections of these sensory cranial nerves are ordered by their developmental sequence, and SAG neurons project to a ventromedial area of the hindbrain, synapsing next to the trigeminal neurons and lateral line neurons (Zecca et al., 2015). The morphological organization of the SAG seems to be preserved in the hindbrain vestibular nuclei (Zecca et al., 2015). The anterior and posterior compartments of the SAG do not intermingle but project to distinct areas. The neurons in the anterior SAG, which innervate the utricle, project to ventromedially relative to neurons in the posterior SAG, which innervate the saccule (Zecca et al., 2015). In addition to central targets, utricular afferents have been observed to synapse on semicircular canal afferents and other utricular afferents (i.e., axo-axonic contacts), which may have modulatory effects (Jia and Bagnall, 2022). Macular SAG organization is maintained in its synaptic targets, which are not limited to the vestibular nuclei.

Few, if any, intracellular recordings have been made of zebrafish primary vestibular afferents. Electrophysiological recordings of second-order vestibular neurons can be used to infer presynaptic activity attributed to first order vestibular afferents (Liu et al., 2020; Hamling et al., 2021). Recordings of excitatory postsynaptic currents (EPSCs) in vestibulospinal (VS) neurons in the hindbrain suggested that multiple primary afferents synapse onto a single VS neuron as demonstrated by different distributions of EPSC amplitudes, frequencies, and directional tuning (Liu et al., 2020; Hamling et al., 2021). Analysis of inter-event intervals of EPSCs indicated the primary afferent input may be irregular in spike timing (Hamling et al., 2021). Although primary vestibular afferent activity has been inferred from secondary neuron recordings, little is known about the electrophysiological properties of SAG neurons and how they compare to other model organisms.

It is unknown whether SAG afferents in zebrafish have similar spiking properties to vestibular afferents recorded in monkeys, rats, mice, frogs, and toadfish (Fernandez and Goldberg, 1976; Baird and Lewis, 1986; Boyle and Highstein, 1990; Kalluri et al., 2010; Songer and Eatock, 2013). In these other organisms, two classes of afferents are present: striolar-synapsing afferents with phasic firing and irregular spike timing and peripherally synapsing vestibular afferents with tonic firing and regular spike timing. These firing properties arise from a multitude of factors, including number of synaptic contacts (few vs. many, respectively), shape of synaptic endings (calyx vs. bouton), size of dendritic arbors (condensed vs. extensive), and complement of ion channels present in cell membranes. The differences in spike timing regularity are known to represent different sensory encoding strategies best suited for distinct ranges of sensory information (i.e., high vs. low frequencies) (Jamali et al., 2016; Curthoys et al., 2017).

Results from Tanimoto et al., 2022 indicate that striolar hair cells and interiorly located neurons in the SAG (which, according to Tanimoto et al., 2022; Liu et al., 2022, synapse on striolar hair cells) have a strong response preference to high frequency, dynamic stimulation. This functional data in zebrafish aligns with responses seen in striolar Type I hair cells and calyx-ending irregular-spiking afferents in mammals, in which this channel is specialized for encoding and transmitting high frequency sensory information (Songer and Eatock, 2013; Jamali et al., 2016; Curthoys et al., 2017). There are several key differences in the afferents in this channel: in zebrafish, there are no calyx endings and no known differences in number synaptic endings when compared to the extrastriola (Liu et al., 2022), although this has not been investigated in adult fish. Whether there is any difference in the distribution of voltage-gated ion channels that contribute to differences in spike timing (Kalluri et al., 2010) is also unknown. Tanimoto et al., 2022 also showed that extrastriola hair cells and their synapsing afferents prefer static tilts (although not exclusively), which also reminiscent of low frequency-preferring regular-spiking afferents in the periphery seen in mammals. Thus, emerging evidence from Ca^2+^ imaging and EM analysis indicates that two parallel channels of vestibular sensory encoding may exist in zebrafish as in mammals, but remains to be resolved.

### Second-order vestibular neurons

**FIGURE 2:**
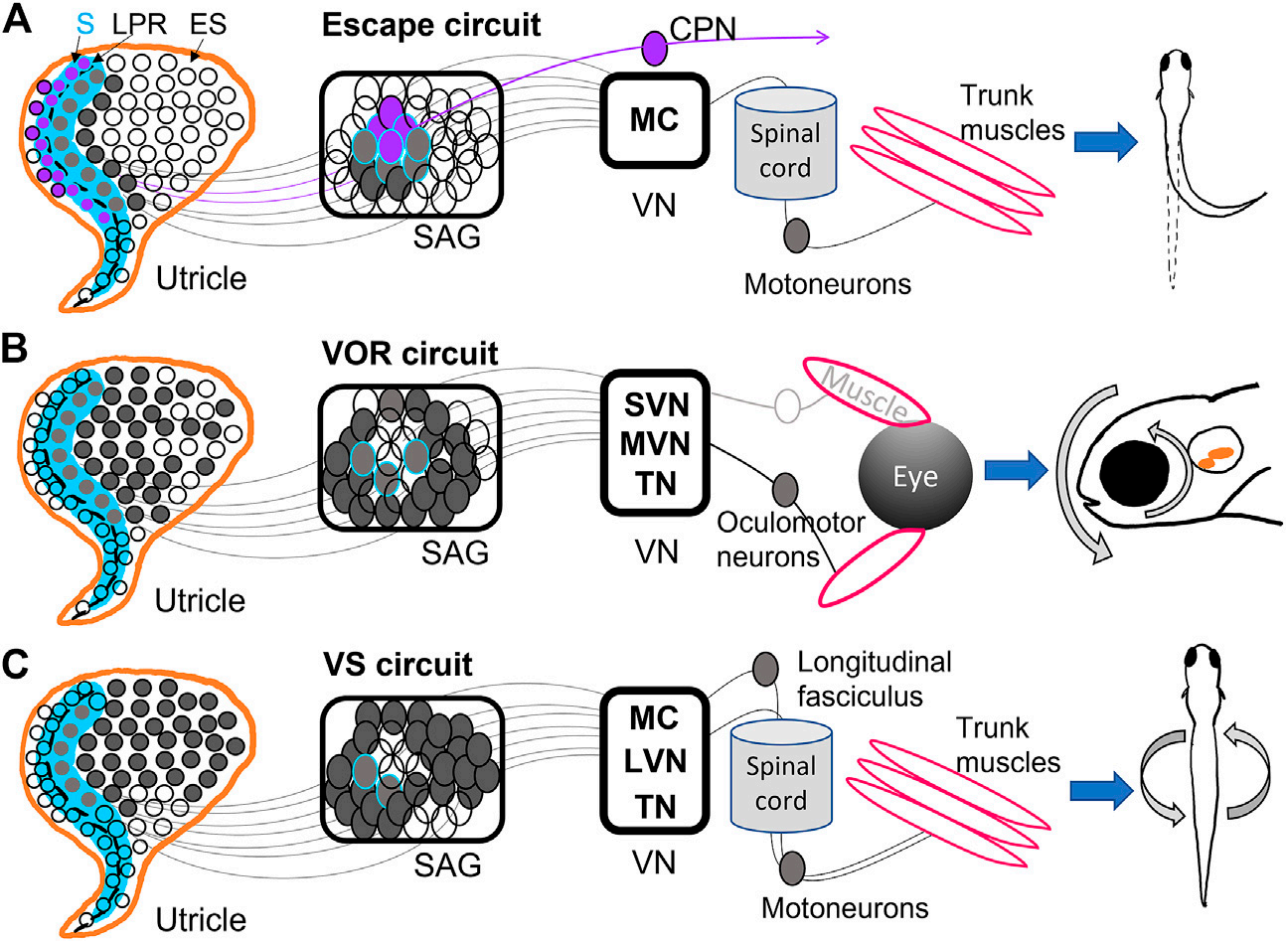
Diagrams of utricular input to vestibular reflexes. **(A)** The Mauthner escape circuit has input from primarily the striolar (S) zone (blue) of the utricle, including the most mature hair cells from both sides of the line of polarity reversal (LPR) (dashed black line). These hair cells are innervated by neurons located in the interior of the SAG, which synapse onto Mauthner cells (MC) in the hindbrain. The ipsilateral MC projects to the spinal cord and motoneurons, among other targets, and ultimately trigger an escape reflex. Hair cells in the lateral utricle across the LPR (purple) have the opposite tuning from those in the medial utricle, and are innervated by SAG neurons that project to commissural-projecting neurons (CPN) that synapse on contralateral MC. Thus, the contralateral MC has opposite directional tuning from the ipsilateral MC (Medan and Preuss, 2014; Bagnall and Schoppik, 2018; Liu et al., 2022). **(B)** The vestibulo-ocular reflex (VOR) circuit receives input from the medial utricle, mostly extrastriolar (ES) hair cells innervated by SAG neurons along the outer shell of the ganglion. These synapse into superior (SVN) and medial (MVN) vestibular nuclei and tangential nucleus (TN). SVN, MVN, TN project contralaterally to oculomotor neurons controlling eye muscles, which result in gaze stabilization (Schoppik et al., 2017; Bagnall and Schoppik, 2018; Liu et al., 2022; Goldblatt et al., 2023). **(C)** The vestibulospinal (VS) circuit also primarily receives input from the medial utricle, and in greatest proportion from ES hair cells and sends projections to MC, TN, and lateral vestibular nuclei (LVN). These VS neurons project to the longitudinal fasciculus or straight down the spinal cord, where motoneurons are activated and posture stabilization initializes (Hamling et al., 2021; Liu et al., 2022; Sugioka et al., 2023).

Much of recent work has focused on circuit development and function of second-order vestibular neurons located in central vestibular nuclei (VN) in the hindbrain (Figure 2) (Schoppik et al., 2017; Liu et al., 2020; Hamling et al., 2021; Liu et al., 2022; Jia and Bagnall, 2022; Goldblatt et al., 2023). Zebrafish have various vestibular nuclei in rhombomeres 5 through 7 of the hindbrain, including the lateral (LVN), medial (MVN), superior (SVN), and descending (DVN) vestibular nuclei and the tangential vestibular nucleus (TN) (Suwa et al., 1999; Highstein and Holstein, 2006; Bianco et al., 2012; Schoppik et al., 2017), as well as other inner ear targets like Mauthner cells (MCs) in rhomobomere 4. These neurons receive input from primary vestibular afferents and drive many important vestibular reflexes, including Mauthner escape circuits, vestibulo-ocular reflex (VOR) circuits, and vestibulospinal (VS) postural circuits (Figure 2).

Mauthner escape circuits drive startle responses and fast escape reflexes based on sensory input from the ear and eye (Sillar, 2009; Medan and Preuss, 2014; Lacoste et al., 2015). MCs are reticulospinal neurons that develop by 8 hpf (Mendelson, 1986) and incite escape behaviors by 1 dpf (Saint-Amant and Drapeau, 1998). They receive direct input from the saccule and utricle and project to locomotor targets (Figure 2A) (Zottoli and Faber, 1979; Szabo et al., 2007; Liu et al., 2022). MCs EM analysis in 5 dpf zebrafish shows that ipsilateral and contralateral MCs receive inputs of opposite direction selectivity from the utricle (Figure 2A) (Liu et al., 2022). MCs ipsilateral to a given utricle receive input from the medial and striolar portion of the macula (Liu et al., 2022). Brainstem neurons that project across the midline (referred to as commissural-projecting neurons, or CPNs) to make contact on the contralateral MC receive input from lateral striola of the utricle, such that each target has opposite tuning (Jia and Bagnall, 2022; Liu et al., 2022) (Figure 2A). This allows for escape reflexes in the direction opposite or away from the detected input. Other subpopulations of CPNs convey both ipsilateral and contralateral tilt from the utricle is across the midline to different contralateral targets (Jia and Bagnall, 2022). Anatomical studies have indicated a direct pathway to MCs from the utricle, which is largely a vestibular organ (Figure 2A) (Szabo et al., 2007; Liu et al., 2022). Although most studies have used acoustic stimuli to incite escape reflexes, Liu et al., 2022 demonstrated that a vestibular stimulus in the form of a translational motion was sufficient to drive escape reflexes, indicating that utricular inputs to MCs are functional. MCs receive input from both ipsilateral and contralateral utricles to that contribute to escape reflex behaviors.

The VOR drives compensatory eye motions in response to head, trunk, or body movements to stabilize gaze (Figure 2B). Early born VOR neurons are observed as early as 30 hpf and continue developing around 48 hpf (Goldblatt et al., 2023). The VOR develops as early as 72 hpf in response to linear translations; angular VOR develops much later, presumably as semicircular canals grow (Beck et al., 2004; Mo et al., 2010; Sun et al., 2018). VOR circuits can originate from neurons in MVN (Schoppik et al., 2017), SVN (Jia and Bagnall, 2022), and TN (Bianco et al., 2012; Goldblatt et al., 2023), which project to oculomotor neurons and generate eye motions that compensate for head movements (Figure 2B). EM reconstruction of VOR neurons in TN and SVN showed inputs from either utricular afferents (Jia and Bagnall, 2022) or semicircular canal afferents (Goldblatt et al., 2023).

VOR neurons in MVN project to ipsilateral oculomotor neurons while VOR neurons in TN project contralaterally (Liu et al., 2022). VOR neurons receiving inputs from the ipsilateral utricle have direction selectivity in either the rostral or caudal directions: each directional subpopulation projects to distinct motor neuron targets which presumably generate eye movements in opposite directions (Jia and Bagnall, 2022). These VOR neurons also display preferential responses to different tilt stimuli: early-born VOR neurons preferentially respond to tilt-up stimuli, while later-born neurons prefer tilt-down (Goldblatt et al., 2023). Indeed, VOR neurons activated via channelrhodopsin drove net downward eye rolls, demonstrating a functional bias to upward (contra-gravity) tilts and thus a strong utricular input in larval fish (Schoppik et al., 2017). Ablation of vestibular neurons in this circuit reduced compensatory VOR following both up and down tilts and hindered swim bladder inflation (Schoppik et al., 2017). VOR neurons take input from both utricular and semicircular canal afferents and drive eye motions with a strong preference for downward motions (Figure 2B).

The VS circuit drives postural and balance reflexes during locomotion (Figure 2C). VS neurons are born between 18 hpf and 32 hpf, well before swimming behaviors initiate at 3 dpf (Hamling et al., 2021). VS neurons located in LVS, MVS, MC, and TN project along the longitudinal fasciculus to motoneurons in the spinal cord (Suwa et al., 1999; Hamling et al., 2021; Sugioka et al., 2023) (Figure 2C). Whole-cell physiological recordings of VS neurons from larval fish revealed complex electrophysiological properties. Hamling et al., 2021 showed that VS neurons had low to no firing at rest, suggesting absence of strong synaptic input at rest, high spiking threshold, and high input resistance. VS neurons fired in response to translational motions during recordings, and displayed directional tuning (Liu et al., 2020; Hamling et al., 2021). Tuning responses of VS neurons are often a summation of multiple synaptic inputs. For example, the majority of afferents converging on VS neurons are similarly directionally tuned, leading to a single preferred direction (Liu et al., 2020; Liu et al., 2022). A minority of VS neurons have complex spatiotemporal tuning likely due to convergence of differently tuned afferents (Liu et al., 2020). Evidence also suggests both electrical and chemical first-order afferent synapses are found on VS neurons (Liu et al., 2020). Recordings after inner ear lesions demonstrated bilateral utricle input to individual VS neurons: high-amplitude EPSCs decreased after ipsilateral lesion while low-amplitude EPSCs decreased after contralateral lesions. VS neuron firing was also reduced in otolith deficient mutants and lesioned fish (Hamling et al., 2021). VS neurons are located across many vestibular nuclei, have both utricular and semicircular input, and have complex electrophysiological properties.

Within the brainstem, development of second-order central vestibular neurons has a spatiotemporal pattern of progression. Liu et al., 2022 found via EM reconstruction that in a larval zebrafish utricle, early born hair cells preferentially signal via early born afferents to early born brain stem populations. The most mature hair cells in the striola of the utricle sent most of their input to MCs, whereas VOR and VS circuits receive input from primarily extrastriolar hair cells (Figure 2A). Optical retrograde labeling of axonal projections identified afferents from the semicircular canals were confined on the later-born VOR neurons in TN (Goldblatt et al., 2023) (Figure 2B). Postural and balance reflexes in the VS circuit follow later as swimming behaviors develop (Figure 2C). The developmental sequence of vestibular sensory epithelia and their input to nuclei has interesting implications for the development of vestibular behaviors: fast escape reflexes develop first with input from the central utricle (Lacoste et al., 2015; Liu et al., 2022), and are later followed by fine balance and postural control.

### Vestibular behaviors and whole brain imaging

Zebrafish posture and balance has been proven to be disrupted by genetic or physiological interventions in the ear and the hindbrain. There are many well documented zebrafish genetic mutations with clear vestibular phenotypes (see Whitfield et al., 1996; Nicolson et al., 1998; Nicolson, 2005; Sheets et al., 2021). Vestibular-deficient fish swim in a spiral, spin, flip upside down, or tip headfirst (Bagnall and Schoppik, 2018). Zebrafish larvae learn to swim in bouts by 4 dpf (Drapeau et al., 2002), and, as they grow, they learn to counterbalance instability with swim motions. Kinematic studies of developing swim behaviors show how swimming coordination changes as larval fish grow: as fish develop, fin and body movements synergize to allow better postural control (Ehrlich and Schoppik, 2019). Fish missing their utricular otolith (*otog*−/− mutants) do not display swimming coordination and cannot keep themselves upright (Bagnall and McLean, 2014; Ehrlich and Schoppik, 2019); although, vestibular input is not necessary for the correct formation of spinal circuits (Roberts et al., 2017). Ablation of neurons in vestibular nuclei also altered swimming behaviors, prevented inflation of swim bladders, and eliminated the corrective movements needed for balance (Schoppik et al., 2017; Hamling et al., 2021). For an in depth look at the development of zebrafish vestibular behaviors and their circuits, see Ehrlich and Schoppik, 2018 and Bagnall and Schoppik, 2018.

Recent studies have reported whole brain calcium imaging responses of vestibular stimulation in larval zebrafish (Favre-Bulle et al., 2017; Favre-Bulle et al., 2018; Migault et al., 2018 Tanimoto et al., 2022; Hamling et al., 2023; Sugioka et al., 2023). Development of new methods to pair imaging with translation and rolling motions have allowed these groups to assess vestibular input to multisensory process and reflexive behaviors. Migault et al., 2018 found vestibular responses throughout the brain in response to sinusoidal rolls in both directions, including the telencephalon, habenula, thalamus, tectum, tegmentum, cerebellum, and medulla. Areas in the hindbrain rhombomeres, possibly VN, and the cerebellum often only responded to motion in one direction (Migault et al., 2018). Although fish were anaesthetized during imaging, it is possible this activity is representative of vestibular processing during behavior. Indeed, Migault et al., 2018 showed that some brain activity was linearly correlated with the posture and velocity of the fish. Favre-Bulle et al., 2017 used optical trapping of otoliths to induce vestibular behaviors from fictive stimuli in larval fish and observed compensatory tail and eye movements from the perceived acceleration and roll. Using this method of stimulation, they reported activity in the VN and premotor activity in the nucleus of the medial longitudinal fascicle (i.e., reticulospinal neurons) (Favre-Bulle et al., 2018). Sugioka et al., 2023 showed that when zebrafish righted their posture after being rolled, neurons in TN and medial longitudinal fasciculus were recruited during the VS reflex. These studies demonstrate that it is possible to characterize complex and multimodal vestibulomotor processing across multiple neural areas involved in posture and balance.

## Summary and future directions

Recent work has made great strides in explaining sensory processing in peripheral and central vestibular circuits. EM reconstruction of the utricle, ganglion, and hindbrain synaptic targets has illustrated the spatiotemporal organization of the developing vestibular system. Striolar and extrastriolar hair cells preferentially respond to vibrations or static tilts and seem to synapse onto different vestibular afferent neuron populations. Afferents, which innervate a single side of the LPR, are also topographically organized in their ganglion and develop in a spatiotemporal manner. Vestibular nuclei may preferentially receive their input from different epithelia zones: escape circuits receive inputs from early developing striolar hair cells while VS and VOR circuits receive input from later developing extrastriolar hair cells and cristae hair cells. Advances in recording technology that allows for calcium imaging during vestibular stimulation has showed whole brain activity during motion, and activation of VOR and VS circuits during vestibular reflexes.

However, understanding of neuronal development and synaptic architecture remains incomplete. How and why are striolar and extrastriolar hair cells in zebrafish preferentially responsive to different stimuli? What kind of signaling prevents afferent neurons from synapsing on both sides of the LPR? What are the constitutive properties of different vestibular afferents that allow them to be better suited to for different kinds of sensory information? How does information propagate from hair cells through vestibular nuclei in the brainstem to forebrain areas? How is directional tuning distributed across brain areas, and are directional asymmetries in vestibular nuclei inherited? How are these circuits assembled during development?

Finally, most, if not all, of vestibular research in zebrafish has been largely restricted to larval and juvenile fish. Some vestibular mutants with strong phenotype deficits (e.g., circling) do not survive past 8 pdf (Riley and Moorman, 2000). The inner ear becomes less accessible as bone hardens and tissues become opaque with age. But research indicates that the vestibular system continues to develop and change as the fish grows in adulthood (Ehrlich and Schoppik, 2019; Schwarzer et al., 2020). Investigation of adult vestibular hair cells, afferents, and circuits, in addition to the current neurobiological research in larval and juvenile fish, will complete our understanding of mature vestibular function.
